# Physical Fitness Levels Do Not Affect Stress Levels in a Sample of Norwegian Adolescents

**DOI:** 10.3389/fpsyg.2017.02176

**Published:** 2017-12-13

**Authors:** Berit Østerås, Hermundur Sigmundsson, Monika Haga

**Affiliations:** ^1^Department of Neuromedicine and Movement Science (INB), Norwegian University of Science and Technology, Trondheim, Norway; ^2^Department of Psychology, Norwegian University of Science and Technology, Trondheim, Norway

**Keywords:** adolescents, perceived stress, physical fitness, mindfulness, pain, BMI

## Abstract

Physical inactivity, low physical fitness, and perceived stress during adolescence are presumed to be risk factors for various disorders and subjective health complaints. On the other hand, physical activity and physical fitness, as well as mindfulness qualities, are regarded as prerequisites for health and well-being in children and adolescent, possibly by moderating the negative effects of stress and protecting against stress-related health complaints. Previous studies have suggested gender differences in the relationship between physical activity/physical fitness and psychological variables. The main objective in this study was to evaluate how physical fitness, along with mindfulness qualities (MAAS-A), pain, and BMI, relate to stress (PSQ) in adolescents. Secondary objectives were to explore the relationship between physical fitness, mindfulness (MAAS-A), and BMI more explicitly in the study sample, as well as to evaluate possible gender differences. The cross-sectional sample included 102 Norwegian pupils in 10th grade (15 or 16 years). Study measurements were four items from the Test of Physical Fitness (TPF), the Norwegian version of the four-factor Perceived Stress Questionnaire (PSQ), the Norwegian version of the Mindful Attention Awareness Scale-Adolescent (MAAS-A), and BMI (recorded in terms of self-reported height and weight). Additionally, pain was measured in terms of localization, number of pain sites, duration, and intensity (Visual analogue scale; VAS). According to the regression analyses, physical fitness could not explain any variation in stress among the adolescents. Nevertheless, there were some negative associations between one stress factor (lack of joy) and components of physical fitness at a group level, possibly influenced by conditions not measured in this study. As opposed to physical fitness, mindfulness qualities, and to some degree gender, seemed to explain variation in stress among the adolescents. None of the physical fitness components were associated to mindfulness (MAAS-A), but some components seemed negatively related to BMI, particularly among the males. Among the females, higher physical fitness (in terms of endurance) seemed related to reduced number of pain sites. Of note, the cross-sectional design did not allow us to determine any causal direction among the variables.

## Introduction

Physical inactivity, low physical fitness, and perceived stress during childhood and adolescence are presumed to be risk factors for various chronic conditions including cardiovascular diseases (Petersen et al., 2012; Bergh et al., 2015), depressive symptoms (Motl et al., 2004; Brunet et al., 2013), pain and subjective health complaints (Sundblad et al., 2008). In turn, physical fitness and physical activity are presumed to moderate the negative effects of stress and protect against stress-related health complaints in children and adolescents (Haugland et al., 2003; Hegberg and Tone, 2015; Gerber et al., 2017a). Gerber et al. (2017a) recently revealed that children (6- to 8-year-old) with higher physical fitness levels, in terms of cardiorespiratory fitness (measured by 20 m shuttle run test), experienced higher levels of psychological well-being relative to their less fit peers when exposed to elevated stress levels (parental questionnaire), and higher levels of physical wellbeing when stress levels were low. Furthermore, children with higher physical activity, as reported by their parents, demonstrated better overall health-related quality of life (parental questionnaire), but only if they experienced low stress (Gerber et al., 2017a). Recently, Cowley et al. (2017) confirmed an inverse relationship between total self-perceived stress and physical activity during spare time in adolescents.

Although strongly related, apparently (Caspersen et al., 1985; Tittlbach et al., 2017), physical activity and physical fitness are terms that describe different concepts, and which are defined and distinguished for health-related research: Physical activity is any bodily movement produced by skeletal muscles that results in energy expenditure. Physical fitness is a set of attributes that people have or achieve, and the health-related components of physical fitness are (a) cardiorespiratory endurance, (b) muscular endurance, (c) muscular strength, (d) body composition, and (e) flexibility (Caspersen et al., 1985). Of note, physical activity during childhood and adolescence seems only weakly related to cardiorespiratory fitness (a) in terms of peak oxygen uptake (Armstrong, 2013). According to a transactional model of stress, stress is experienced when a person perceives that the demands overload or exceed the personal and social resources the individual is able to mobilize (Lazarus and Folkman, 1984).

In particular, cardiorespiratory fitness is proposed to protect against health-threatening reactions to acute psychosocial stress (Wyss et al., 2016; Gerber et al., 2017a), and stress-related symptoms of burnout and depression (Gerber et al., 2013a,b; Elliot et al., 2015). Recently, authors have recommended vigorous physical activity in adolescents to prevent burnout symptoms in this group, based on the demonstrated negative correlations between these variables (Gerber et al., 2013b; Elliot et al., 2015). Cardiorespiratory fitness is also proposed to protect against overweight and adiposity in children, adolescents, and young adults (Gerber et al., 2017b; Hingorjo et al., 2017). Gerber et al. (2017b) found that children (7–8 years) experiencing elevated school-related stress had lower body mass index (BMI), body fat, and waist circumferences if they had high physical fitness (measured by 20 m shuttle-run test) and physical activity levels.

In general, physical activity and physical fitness are considered beneficial in adolescents, resulting in consensus statements and activity guidelines for this group (Hallal et al., 2006; Tremblay et al., 2011). Several of the health beneficial effects, with respect to various subjective health complaints including pain and psychological symptoms, apparently relate to reduced stress or stress resilience (Haugland et al., 2003; Sundblad et al., 2008; Moljord et al., 2014; Bergh et al., 2015; Hegberg and Tone, 2015; Gerber et al., 2017a). Mindfulness qualities also seem related to reduced stress in adolescents (Bluth et al., 2015; Galla, 2016). Moreover, studies have shown that physically active lifestyles are related to better mindfulness skills (Kangasniemi et al., 2014), and that mindfulness possibly mediates the beneficial (stress resilient) effects of physical fitness (Demarzo et al., 2014). Mindfulness is typically described as a form of nonjudgmental, nonreactive attention to present moment experiences, both internal and external (Brown and Ryan, 2003; Kabat-Zinn, 2003).

Hence, it might be reasonable to assume that physical fitness will relate negatively to perceived stress and pain and positively to mindfulness in adolescents. However, this is not previously investigated in Norwegian adolescents. Previous studies have demonstrated that resilience, which seems relevant for the positive effects of physical activity and physical fitness in adolescents, associates negatively with perceived stress (Montero-Marin et al., 2014) and positively with mindfulness qualities (Montero-Marin et al., 2015). Furthermore, previous studies have suggested plausible gender differences in the relationship between physical activity/physical fitness and psychological variables (Moksnes et al., 2014; Moljord et al., 2014), which are relevant to investigate further.

The main objective in this study was to evaluate how physical fitness, along with mindfulness qualities, pain, and BMI, relate to perceived stress in adolescents. Secondary objectives were to explore the relationship between physical fitness, mindfulness, and BMI more explicitly in the study sample, as well as to evaluate possible gender differences.

## Materials and methods

### Design of the study

This was a cross-sectional study.

### Sample and settings

The sample included 102 Norwegian pupils in 10th grade (15 or 16 years), recruited from a public school in the Trondheim municipality. The participating school was considered to be an “average” school regarding sociocultural- and economic conditions (Ownership Unit in Trondheim Commune, 2012).

### Sample size

Sample size calculation (power analyses) showed that 90 participants were required to have 84% power to significantly detect a correlation (*r*) of 0.30 in the population at the 5% level (standard error 0.10, 95 % CI: 0.12–0.46). This number was increased to 110 to allow for withdrawals. The total number that completed both questionnaires and physical fitness test were *N* = 102, giving an overall response rate of 92.7%. 62 (60.8%) females and 40 (39.2%) males were included. The data was collected autumn 2015.

### Variables and instruments

#### The perceived stress questionnaire (PSQ)

The recent form of the 30-item PSQ, which is used in this work, refer to the period of the last 4 weeks and can be answered with a 4-point rating scale (1 = almost never, 2 = sometimes, 3 = often and 4 usually; Levenstein et al., 1993; Fliege et al., 2005; Kocalevent et al., 2007). Higher scores indicate more severe perceived stress. The resulting PSQ total score is linearly transformed between zero and one; PSQ = (raw value – 30)/90 (Levenstein et al., 1993). The cut-off score for moderate to severe stress is set to ≥0.45, based on the mean PSQ scores in the present study samples, which corresponds with previous findings and practice (Kocalevent et al., 2007, 2011). Commonly used cut-off levels of stress with respect to the PSQ are low <0.33; medium ≥0.33 <0.45; moderate ≥0.45 <0.60; severe ≥0.60. Scale instructions are “For each sentence, circle the number that describe how often it applies to you in general, during the last month. Work quickly, without bothering to check your answers, and be careful to describe how it applies to you *in general*.” The PSQ permits the subjective experience of perceived stressful situations and stress reactions to be assessed, emphasizing cognitive perceptions more than emotional states or specific life events, and is considered a reliable and valid instrument for recording perceived stress in the context of a transactional view of stress (Kocalevent et al., 2007). It contains both positively and negatively formulated items in order to reduce acquiescent bias. Examples of positively formulated items are “You feel rested,” “You are full of energy,” and “You enjoy yourself.” Examples of negatively formulated items are “You feel that too many demands are being made on you,” “You have too many things to do,” and “You have many worries.” Each item is answered using a four-point Likert-type scale, ranging from 1 (“almost never”) to 4 (“almost always”). The translation process and psychometric evaluation of the Norwegian version of the PSQ are explained in detail in a separate paper, providing support for a four-factor model [“worries,” “tension,” (lack of) “joy,” “demands” (Fliege et al., 2005)], of the PSQ in Norwegian adolescents (Østerås et al., in review).

#### Physical fitness

To measure physical, four items from the Test of Physical Fitness (TPF) (Fjørtoft et al., 2011) were used for testing strength, speed, agility/plyometrics, and endurance, primarily. The four test items are as follows:

1. Pushing a medicine ball (2 kg) with two hands as far as possible. The starting position is with the feet parallel to each other and shoulder width apart, with the ball held against the chest. The test item score (better of two attempts) is the distance achieved (measured in meters).
2. Running 20 m as quickly as possible. The participant starts in a standing position. At signal, the participant runs as fast as possible toward the finish line. The test item score is the time in seconds needed to run the 20 m.
3. Standing broad jump. The participant starts with the feet parallel and a shoulder width apart behind a starting line. At a signal, the participant swings his or her arms backward and forward and jumps with both feet simultaneously as far forward as possible. The test item score (the better of two attempts) is the distance (in centimeters) between the starting line and the landing position.
4. Reduced Cooper Test. The participant runs or walks around a marked rectangle measuring 9 × 18 m (the size of a volleyball field) for 6 min. Running and walking are allowed. The test item score is the distance covered (in m) in 6 min.

All test item scores were transformed into standardized score (z-scores) from the mean of the whole sample (*n* = 102). The score of test item 2 (running 20 m as quickly as possible), which measured the time needed to accomplish the test item, was beforehand converted to 1/score, such that higher scores always indicated better performance than lower scores. The test items scores represent subcategories or “sub-variables” of physical fitness, involving different components of physical fitness. Higher z-scores indicate better performance on the tasks. A total test score was calculated to express the adolescent's overall performance on the four physical fitness measures, as a “sum-variable” of total physical fitness. The total test score for each participant was defined as the average z-score on all test items successfully performed by that individual. Fjørtoft et al. (2011) have previously evaluated the TPF in Norwegian children, finding high internal consistency and convergent construct validity as well as fair to good test-retest reliability for individual test items scores and total score. Items from the TPF are also previously applied in adolescents (Gísladóttir et al., 2013).

#### The mindful attention awareness scale for adolescents (MAAS-A)

The MAAS-A includes 14 items, where the responses are made on a 6-point scale, where higher scores reflect more mindfulness-capacity, regarding the addressed quality (Brown et al., 2011; Quaglia et al., 2016). The test-authors have recently specified that the MAAS or MAAS-A focus on a quality of attentiveness involved in mindfulness (Quaglia et al., 2016). Scale instructions were “Below is a collection of statements about your everyday experience. Using the 1–6 scale below, please indicate how frequently or infrequently you currently have each experience. Please answer according to what really reflects your experience rather than what you think your experience should be.” The accompanying 6-point scale was 1 (almost always), 2 (very frequently), 3 (somewhat frequently), 4 (somewhat infrequently), 5 (very infrequently), 6 (almost never). The total sum score of the MAAS-A is the computed mean of the items scores. The authors of the MAAS-A granted us permission for translation and back translation of the MAAS-A, and authorized our final version. The translation process of the instrument into Norwegian is explained in detail in another paper (Østerås et al., in review), wherein the instrument also was evaluated in relation to stress (PSQ). Another recent study has also provided support for the one-factor model of the Norwegian MAAS-A (short-form), with respect to model-based reliability, measurement invariance and concurrent validity (Smith et al., 2017).

#### Pain

Pain was measured by questions about localization (pain site), duration, and intensity. Pain site (0–6) was divided into six main categories, corresponding to the questionnaire in the Young-HUNT Study 2008 (Hoftun et al., 2011; Skrove et al., 2015); head, neck, shoulder, back, arm, lower extremity, with an open line for additionally sites. Three or more (≤3) pain sites were termed multisite pain. Pain duration (1–5) was divided into five categories; 0–2 weeks, 2–4 weeks, 1–2 months, 2–3 months, 3 months, or more. Pain intensity (Visual analog scale VAS: 0–10) was measured using a VAS-line (Price et al., 1983). The participants were instructed to mark on the VAS-line (10 cm) to illustrate their average pain during the last week, with 0 indicating “no pain” and 10 “worst pain imaginable.” VAS is described and applied as an acceptable measurement of average pain during the last week (Breivik et al., 2008; Bäckryd et al., 2016). VAS is demonstrated to be reliable as a measure of pain intensity in children (Bailey et al., 2012) and is used to detect differences in pain between groups of adolescents (Sugiura et al., 2015). The questionnaire also asked if former injuries, diseases or disorders might be related to the current pain experiences.

### Body mass index

BMI was also recorded, in terms of self-reported height and weight, as this health variable is regarded relevant in relation to the other outcome measures (Sethi et al., 2011; Silva et al., 2014; Loucks et al., 2015; Gerber et al., 2017b).

Variables, instruments, and measurements are presented in Table 1.

**Table 1:** Variables and instruments.

**Variables**	**Instruments/measurements**
**Stress** Worries Tension Joy (lack of) Demands	The Perceived Stress Questionnaire (PSQ)
**Physical fitness** Pushing a medicine ball Running 20 m quickly Standing broad jump Reduced Cooper Test	The Test of Physical Fitness (TPF)
**Mindfulness**	The Mindful Attention Awareness Scale Adolescent (MAAS-A)
**Pain** Location Number of pain sites Pain duration Pain intensity	Questionnaire Visual Analogue Scale; VAS
**Body mass index (BMI)** Height Weight	Questionnaire

### Ethics and procedures

The Regional Committee for Medical Research Ethics in Trondheim approved the data collection processes and the entire study, and the study was in line with the Declaration of Helsinki (The World Medical Association (WMA), 2013). The purpose of the study, the outcome measures and the procedures were explained and in consensus with the participating school (principal and teachers). The adolescents and the parents received an information letter that briefly explained the purpose of the study. In all stages of the data collection, it was emphasized that participation was voluntary, anonymous, and confidential, and that the participants were free to withdraw from the study at any point without giving a reason. Informed consent was obtained from all participants. Students already 16 years were responding on their own behalf. Students not yet turned 16 at the time of data collection received a written consent from their parents. Participating in the study was not presumed to affect the students in any negative way.

The questions are addressing everyday experiences/situations. The physical fitness test contains well-known motor fitness tasks, commonly applied in school gymnastics. The assessment of the physical fitness took place in a sport hall during school hours, in line with previously applied protocols (Haga et al., 2015). Assistants trained in the test protocols tested all the participants individually. Each test item was explained and demonstrated before the participants started. The participants were given verbal encouragement and support throughout the testing procedure. If the participants made a procedural error, instructions and demonstrations were repeated, and the participants made a new attempt. The participants wore clothing suitable for physical activity and sport shoes during both tests. Identification numbers were used for the participants in the study to maintain data confidentiality. The participants accomplished the test items individually in groups, performing different tasks simultaneously. Thus, no one was competing against each other, and no one (of the participants) was watching others completing the test. Test results were filled into a form by the administrator; on page 1 (behind a neutral front page) of a questionnaire folder, and immediately after handed to the participant for completion of the questionnaire on page 2 and 3 in the questionnaire folder. After answering the questionnaire in a silent and sheltered place, the participant enclosed the questionnaire folder in an envelope. The administrator collected the concealed envelopes.

### Statistical analysis

The statistical analyses were conducted in IBM SPSS 24. Preliminary analyses were performed to check for univariate and multivariate normality. Descriptive analyses of the outcome variables were calculated, also separately for gender. Spearman's rank correlation coefficient (*r*_*s*_) was used to assess the associations between the continuous variables (due to lack of normality of pain variables), also with a split file command with respect to gender. Linear regression analyses evaluated the probability that physical fitness, mindfulness, and pain (duration) in addition to gender and BMI could explain variation in stress (lack of joy), with the latter variable applied as the dependent variable. The linear regression model was developed backward, in order to minimize suppressor effects and to reduce the risk of making a Type II error (Field, 2013, pp. 323–324).

## Results

Table 2 summarizes the descriptive analyses of dichotomous variables, overall and separately for gender. 52.5% of the males and 45.2% of the females reported pain (Table 2). The prevalence of head pain/headache was highest among the females (Table 2). A similar portion of females and males related their pain to former injuries (25.8% of the females and 27.5% of the males) or to a known disease or disorder (9.7% of the females and 7.5% of the males). The prevalence of moderate to severe stress (PSQ ≥ 0.45) seemed slightly higher among the females, i.e., 27.4% of the females against 20% of the males reported PSQ ≥ 0.45. Descriptive analyses of the continuous variables are presented in means and standard deviation (SD) in Table 3, both overall and separately for gender. Higher scores on the physical fitness measures (except from sprint), indicate higher physical fitness. Higher scores on the PSQ, including factors of PSQ, indicate higher stress. Higher scores on the MAAS-A reflect more mindfulness-capacity with respect to the addressed mindfulness-quality. The pain variables (particularly pain intensity and pain sites) were not normally distributed; hence, pain descriptions in the subsamples of females and males reporting pain are also included in Table 3.

**Table 2:** Descriptive analyses of dichotomous variables, presented in percentage (%).

	**Overall (*N* = 102)**	**Females (*n* = 62)**	**Males (*n* = 40)**
**Pain**			
Have pain	48.0	45.2	52.5
Head pain	12.8	17.7	5.0
Neck pain	5.9	6.5	5.0
Shoulder pain	2.9	4.8	0.0
Back pain	16.7	17.7	15.0
Arm pain	2.9	3.2	2.5
Leg pain	28.4	22.6	37.5
Former injury related to pain	26.5	25.8	27.5
Known disease related to pain	8.8	9.7	7.5
**Stress**			
Moderate to severe stress (PSQ ≥ 0.45)	24.5	27.4	20.0

*PSQ, Perceived stress questionnaire*.

**Table 3:** Descriptive analyses of the continuous variables presented as means (SD).

	**Overall (*N* = 102)**	**Females (*n* = 62)**	**Males (*n* = 40)**
**Stress (total PSQ; 0–1)**	0.34 (0.16)	0.37 (0.16)	0.29 (0.15)
Worries	0.33 (0.22)	0.35 (0.22)	0.30 (0.22)
Tension	0.36 (0.18)	0.40 (0.19)	0.29 (0.15)
Joy (lack of)	0.34 (0.17)	0.40 (0.16)	0.26 (0.17)
Demands	0.48 (0.21)	0.53 (0.19)	0.40 (0.21)
**Mindfulness (MAAS-A; 1-6)**	4.05 (0.80)	4.03 (0.82)	4.09 (0.76)
**Physical fitness**			
Total test score (based on z-scores)	–	−0.26 (0.65)	0.38 (0.72)
Pushing a medicine ball (m)	5.63 (1.15)	4.99 (0.69)	6.69 (1.04)
Running 20 m (sec)	4.04 (0.29)	4.16 (0.28)	3.87 (0.24)
Standing broad jump (m)	1.70 (0.26)	1.57 (0.22)	1.91 (0.20)
Reduced Cooper test (m)	1071.27 (175.66)	1005.64 (163.77)	1171.26 (146.35)
**BMI**	20.25 (2.05)	20.06 (2.20)	20.63 (1.85)
**Pain**		**(***n*** = 28)**	**(***n*** = 21)**
Pain sites (0–6)	0.69 (0.93)	1.61 (0.96)	1.24 (0.54)
Duration (1–4)	2.0 (1.36)	3.41 (1.05)	2.70 (1.38)
Intensity (VAS; 0-10)	1.8 (2.40)	4.34 (2.38)	2.86 (1.56)

*PSQ, Perceived stress questionnaire; MAAS-A, Mindful Attention Awareness Scale - Adolescent; Physcial Fitness, Test of Physical Fitness; Jump, Standing broad jump; Ball push, Pushing a medicine ball; Sprint, Running 20 m as quickly as possible; Red. Cooper, Reduced Cooper test; BMI, Body Mass Index; VAS, Visual analogue scale*.

Spearman's rank correlation coefficient (*r*_*s*_) was used to assess the associations between the outcome variables, since not all (pain) variables were normally distributed (Table 3). Tables 4, 5 summarize the results of the correlation analyses with respect to stress and physical fitness, respectively, both overall and separately for gender.

**Table 4:** Associations (*r*_*s*_) between, stress, physical fitness, pain, mindfulness and BMI, overall, and separately for gender.

		**Physical fitness, total score (z)**	**Ball push**	**Sprint**	**Jump**	**Reduced Cooper**	**Pain sites**	**Pain duration**	**VAS**	**MAAS-A**	**BMI**
Stress	Total PSQ	0.09	−00.04	0.12	0.11	−0.16	−0.01	−0.01	0.00	−0.58^**^	0.19
(overall, *N* = 102)	Worries	0.02	−0.01	0.06	0.09	−0.02	−0.01	−0.05	−0.03	−0.49^**^	0.21^*^
	Tension	0.11	−0.05	0.18	0.12	−0.13	0.08	0.07	0.10	−0.52^**^	0.10
	Joy (lack of)	−0.05	−0.21^*^	−0.01	−0.06	−0.23^*^	−0.12	−0.06	−0.07	−0.33^**^	0.11
	Demands	0.08	−0.06	0.12	0.10	−0.16	0.00	0.02	−0.02	−0.49^**^	0.09
Stress	Total PSQ	0.08	0.13	0.04	0.06	0.10	0.16	0.11	0.17	−0.63^**^	0.22
(females, *n* = 62)	Worries	0.06	0.04	0.01	0.07	0.04	0.15	0.05	0.14	−0.54^**^	0.24
	Tension	0.03	0.11	0.10	0.02	0.05	0.25	0.20	0.27^*^	−0.55^**^	0.10
	Joy (lack of)	−0.11	0.00	−0.09	−0.14	−0.01	0.03	0.09	0.11	−0.37^**^	0.16
	Demands	0.04	0.20	0.03	−0.00	0.12	0.06	0.03	0.02	−0.57^**^	0.10
Stress	Total PSQ	0.06	0.36^*^	0.21	0.16	−0.31	−0.28	−0.25	−0.34^*^	−0.56^**^	0.14
(males, *n* = 40)	Worries	−0.05	0.24	0.14	0.11	−0.31	−0.26	−0.24	−0.32^*^	−0.41^**^	0.19
	Tension	0.14	0.38^*^	0.25	0.23	−0.17	−0.21	−0.28	−0.27	−0.51^**^	0.14
	Joy (lack of)	−0.02	0.17	0.05	−0.02	−0.19	−0.41^**^	−0.48^**^	−0.48^**^	−0.33^**^	0.10
	Demands	0.06	0.31	0.20	0.19	−0.21	−0.11	−0.08	−0.20	−0.44^**^	0.09

* p < 0.05;

** *p < 0.01. PSQ: Perceived stress questionnaire; MAAS-A; Mindful Attention Awareness Scale-Adolescent; BMI: Body Mass Index; VAS: Visual analogue scale; Physcial Fitness: Test of Physical Fitness; Jump: Standing broad jump; Ball push: Pushing a medicine ball; Sprint: Running 20 m fast; Red. Cooper: Reduced Cooper test*.

**Table 5:** Associations (*r*_*s*_) between physical fitness, pain, mindfulness and BMI, overall, and separately for gender.

		**Pain sites**	**Pain duration**	**VAS**	**MAAS-A**	**BMI**
Physical Fitness	Total score (z)	0.01	−0.10	0.03	−0.15	−0.11
(overall, *N* = 102)	Ball push	0.07	0.03	0.06	−0.13	0.19
	Sprint	−0.04	−0.08	−0.02	−0.15	−0.25^*^
	Jump	0.01	0.02	0.07	−0.09	−0.21
	Red. CooperCooper (z)	−0.06	−0.17	−0.04	−0.01	−0.20
Physical Fitness	Total score (z)	−0.07	−0.09	0.01	−0.19	−0.09
(females, *n* = 62)	Ball push	0.15	0.14	0.21	−0.25	0.15
	Sprint	−0.06	−0.07	−0.02	−0.20	−0.19
	Jump	0.05	0.03	0.93	−0.08	−0.08
	Red. Cooper	−0.28^*^	−0.23	−0.15	−0.13	−0.21
Physical Fitness	Total score (z)	0.10	0.04	0.11	−0.04	−0.29
(males, *n* = 40)	Ball push	−0.13	0.09	−0.19	−0.25	0.16
	Sprint	−0.02	−0.11	−0.03	−0.07	−0.35^*^
	Jump	−0.05	−0.04	0.01	−0.10	−0.40^*^
	Red. Cooper	0.21	0.06	0.15	0.19	−0.37^*^

* *p < 0.05. Physcial Fitness, Test of Physical Fitness; Jump, Standing broad jump; Ball push, Pushing a medicine ball; Sprint, Running 20 m fast; Red. Cooper, Reduced Cooper test; MAAS-A; Mindful Attention Awareness Scale-Adolescent; BMI, Body Mass Index; VAS: Visual analogue scale*.

Table 4 shows significant (*p* < 0.001) and strong negative associations between stress and mindfulness, both overall and separately for gender. Among the females, demands appeared to be strongest related (*r*_*s*_ = −0.57) to mindfulness, while tension (*r*_*s*_ = −0.51) appeared strongest related to mindfulness in males.

Joy (lack of) seemed negatively associated (*p* < 0.05) to physical fitness in terms of pushing a medicine ball (strength) and reduced Cooper test (endurance) in the overall sample (Table 4). Also in the overall sample, worries seemed positively related (*p* < 0.05) to BMI.

Among the females, tension seemed positively associated (*p* < 0.05) to pain intensity (measured by VAS). Among the males in the study sample, several of the associations appeared reversed: Both total stress (total PSQ) and tension seemed positively associated (*p* < 0.05) to physical fitness in terms of pushing a medicine ball (strength). Additionally, joy (lack of) appeared negatively associated to pain sites and pain duration, while total stress (total PSQ), worries and joy (lack of) seemed negatively associated to pain intensity (VAS) (Table 4).

Table 5 presents more of the physical fitness associations. In the overall sample, physical fitness, measured by running fast 20 m, was negatively associated (*p* < 0.05) with BMI. Among the females, physical fitness, measured by the reduced Cooper test (endurance), was negatively associated (*p* < 0.005) to number of pain sites. Among the males, physical fitness in terms of running 20 m, standing broad jump and reduced Cooper, were negatively associated (*p* < 0.05) with BMI. There was no associations between physical fitness and mindfulness qualities measured by the MAAS-A (Table 5).

The linear regression analysis with stress (lack of joy) as dependent variable, revealed that mindfulness measured by the MAAS-A (β = −0.40, *p* < 0.001) and gender (PSQ, β = −0.33, *p* < 0.05) explained some of the variation in the stress factor (Table 6). Physical fitness (total test score), pain duration and BMI did not explain any variation in the stress factor, according to the regression analyses. The regression model explained 26.3 % of the variation in the stress factor (lack of joy). The *R*^2^ (0.263) and significant *F*-value (5.36, *p* < 0.001) supported acceptable fit of the model. The adjusted *R*^2^ (0.214) indicated very sparse loss of power. A Durbin-Watson test value close to two (1.70) indicated independence of the residuals.

**Table 6:** Summary of the linear regression analysis for variables predicting joy (lack of joy, as factor of stress [PSQ]).

	**B**	**SE B**	**β**	**F**	***R*^2^**
Constant	0.82	0.23			
Gender	−0.12	0.04	−0.33^*^		
BMI	0.01	0.01	0.05		
Pain duration	−0.02	0.01	−0.12		
Physical fitness	−0.02	0.02	−0.12		
Mindfulness	−0.09	0.02	−0.40^***^		
				5.36^***^	0.26

* p < 0.05;

*** *p < 0.001*.

*BMI, Body Mass Index; PSQ, Perceived Stress Questionnaire*.

## Discussion

In this study, physical fitness along with mindfulness qualities, pain and BMI were evaluated in relation to stress in adolescents. Other physical fitness-associations were also explored in addition to gender differences. According to the main findings, physical fitness cannot explain any variation in stress among the adolescents (Table 6). Nevertheless, there were some negative associations between one stress factor (lack of joy) and components of physical fitness (strength and endurance) at a group level (Table 5), possibly influenced by conditions not measured in this study. As opposed to physical fitness, mindfulness qualities seemed to explain variation in stress among the adolescents (Table 6). Physical fitness seemed entirely unrelated to mindfulness (MAAS-A) but somewhat (negatively) related to BMI, particularly among the males (Table 5). Among the females, higher physical fitness (in terms of endurance) seemed related to reduced number of pain sites (Table 5).

Regression analyses revealed that physical fitness did not explain any of the variation in stress (lack of joy) (Table 6). Hence, the results do not support that physical fitness protects against stress in adolescents. Rather, the results might correspond with the findings by Klaperski et al. (2013, 2014). They revealed unexpected psychological and physiological stress responses to exercises, including higher mood decrease in young women (Klaperski et al., 2013). Mindfulness qualities (MAAS-A), on the contrary, seemed to explain some of the variation in stress (Table 6). This is in compliance with previous findings, showing improved mindfulness qualities to be accompanied by reduced stress in adolescents (Bluth et al., 2015; Galla, 2016). Furthermore, physical fitness seemed unrelated to mindfulness qualities (MAAS-A) among the adolescents (Table 5). In sum, this might imply that the stress-preventive effect of physical fitness rely on mediating factors/variables such as mindfulness qualities, which is also previously suggested (Demarzo et al., 2014), or variables not measured in this study such as coping strategies.

The weak association (*p* < 0.05) between higher physical fitness (strength and endurance) and lower stress (increased joy) at a group level (Table 5) might be influenced by conditions not measured in this study. The finding might also be comparable to the results in Moljord et al. (2011). They found that adolescents who reported physical activity-participation two to three times per week or more scored significantly lower on stress (Norwegian version of the Adolescent Stress Questionnaire) and higher on happiness (Fordyce Happiness Scale) than those who participated in physical activity 1 day per week or less. However, they found no significant difference on stress and happiness between those being physically active two or three times a week and those being active almost every day. This might correspond to the findings by Lutz et al. (2010), revealing a positive relationship between stress and exercise frequency, intensity, and duration in students exercising three times a week or more (for 20 min or more each time and for more than 6 months). Put together, previous and present findings suggest that the health beneficial effects of physical activity and physical fitness probably relay on several factors. Among others, activity dose (volume, frequency, intensity) and the factors that initiates/regulates the activity probably influence the outcome (Moljord et al., 2011, 2014; Sibley et al., 2013; Gerber et al., 2015). Sibley et al. (2013) showed that higher intrinsic motivation (more intrinsic motives and intrinsic forms of motivation) predicted healthier levels of physical fitness in students in contrast to stronger introjected regulation and appearance motives, which predicted worse fitness.

At a group level (overall sample), there seemed to be a weak association (*p* < 0.05) between higher stress in terms of worries and higher BMI (Table 4). This might be considered in compliance with previous studies (Sethi et al., 2011; Gerber et al., 2017b), finding positive associations between stress and BMI. However, this was not evident in the analyses separately for gender. Moreover, higher physical fitness seemed associated (*p* < 0.05) with lower BMI, both in the overall sample and among the males (Table 5). The negative physical fitness-BMI association might be comparable to the findings by Gerber et al. (2017b). They found that children (7–8 years) experiencing elevated school-related stress had lower body mass index, body fat, and waist circumferences if they had high physical fitness (measured by 20 m shuttle-run test) and physical activity levels. Besides, the physical fitness tasks which were associated with reduced BMI in this study, i.e., running 20 m, standing broad jump, and reduced Cooper (Table 5), commonly involve cardiorespiratory training/fitness which associate with lower BMI in adolescents and young adults (Hingorjo et al., 2017). However, the negative physical fitness-BMI association was not possible to identify among the females (Table 5).

Among the females in this study, higher stress in terms of tension seemed associated with more pain in terms of higher pain intensity (Table 4). This corresponds to previous findings in Norwegian adolescents (Østerås et al., 2015, 2016). However, this finding was not evident in the overall sample, nor among the males, possibly due to the non-normal distribution of pain variables in the sample (Table 3). Also among the females, there appeared a negative physical fitness-pain association (*p* < 0.05) (Table 5). More specific, increased endurance (higher scores on the reduced Cooper test) seemed related to reduced number of pain sites (Table 5). At first, this might resemble activity-induced pain modulation or hypoalgesia (Naugle et al., 2013; Jones et al., 2014; Saanijoki et al., 2017). However, the study design does not allow such an evaluation. It is also likely that the females with fewer pain sites just performed better at the test-day. There were no other associations between physical fitness and pain in this study (Table 5). This is in contrast to previous studies, suggesting pain-moderating effects of physical activity and physical fitness in children and adolescents (Haugland et al., 2003; Sundblad et al., 2008; Gerber et al., 2017a). However, these prior results might have been mediated by reduced stress (Haugland et al., 2003; Sundblad et al., 2008; Gerber et al., 2017a).

The demonstrated gender differences in this study, with respect to stress (Table 3) and its' relation to other health variables (Tables 4, 5), might be considered in compliance with previous studies. Several authors have suggested gender differences in stress appraisals, reactions and responses, both on a behavioral and a neurobiological level (Wang et al., 2007; Mayor, 2015; Marrocco and McEwen, 2016; McEwen and Milner, 2017). Furthermore, in the study by Moljord et al. (2014) in Norwegian adolescents (13–18 years old), physical activity associated with psychological symptoms in the females but not in the males. In the present study also, some of the associations seemed reversed among the males. For instance, higher physical fitness (in terms of strength) seemed associated (*p* < 0.05) with higher stress among the males, in terms of total PSQ and tension (Table 4). This positive physical fitness-stress association might be comparable with the findings by Lutz et al. (2010) and Gerber et al. (2015). Gerber et al. (2015) found that exercise involvement was associated with increased stress in adolescents with high exercise self-regulation. The adolescents who accepted to participate in this study, which involved willingness to perform a physical fitness test, probably were relatively high in exercise self-regulation. However, this was not measured. Furthermore, among the males in the study sample there appeared negative associations between some of the stress factors and the pain variables (Table 4). This is in contrast to previous findings in Norwegian adolescents (Østerås et al., 2015, 2016). Probably, the findings in this study sample were influenced by the non-normal distribution of pain variables (Table 3). However, the findings might also imply that perceived stress associate differently with pain in a sample of fit adolescent males (see the “Strengths and limitations”-paragraph) than in the general adolescent population.

The lack of support in this study concerning the presumed stress and pain preventing effect of physical fitness in adolescents, might also be understood in light of modern societal trends and cultures. For instance, the promotion of exercise and physical fitness for appearance-motivated reasons are common in our modern society, extensively conveyed by “fitspiration” websites, which often reinforce an over-valuation of physical appearance, eating concerns, and excessive exercise (Boepple et al., 2016). This might contribute to more unhealthy physical fitness trends and habits, probably with less beneficial health outcomes as a result (Sibley et al., 2013; Cunningham et al., 2016). A recent population-based longitudinal study from Sweden revealed a shift in the covariates of perceived health, from physical fitness (cardiorespiratory) and chronic illness in 1990 to age, BMI, and educational level in 2015, i.e., a diminished impact of physical fitness on perceived health (Olsson et al., 2017).

### Strengths and limitations

One strength of this study is the inclusion of an objective measure of physical fitness in adolescents. This prevents self-reporting bias. Previous studies with similar focus in Norwegian adolescents have been based on self-reported physical activity (Haugland et al., 2003; Moljord et al., 2011, 2014). Self-reported physical activity and objectively assessed physical activity/physical fitness have previously demonstrated to be differently and even inversely related to self-rated health (Hamer and Stamatakis, 2010; Lindwall et al., 2012). Another strength of the study is a recent validation of the included stress-instrument (PSQ) in Norwegian adolescents, providing support for the four-factor model and measurement invariance across gender (Østerås et al., in review).

However, we also acknowledge several limitations. Neither the cross-sectional design nor the correlation analyses are entitled to determine any causal relationships between the variables. Furthermore, the inclusion of a physical fitness test might have caused a selection bias. Adolescents already confident and comfortable in physical performances possibly appreciated the invitation and easily accepted to participate in the study. Less fit adolescents possibly declined to participate at a higher rate than their more fit peers. Adolescents perceiving pain and health complaints probably also declined more frequently that their pain-free (or approximately pain-free) peers. The sudden withdrawals at the day of testing due to pain and other subjective health complaints (voluntarily reported), might indicate such dropout mechanisms. The non-normally distribution of the pain-variables might also be an indicator of this. However, this was not evaluated in more detail. Furthermore, the self-reported height and weight, from which the BMI-values were calculated, were possibly not entirely reliable. In order to explain stress, several other factors and variables are also relevant, such as adverse childhood experiences, coping strategies, and catastrophizing. However, those fall beyond the defined scope of this study. Finally, the sample size was not very large (*N* = 102), and the findings probably needs to be confirmed in further studies.

## Conclusion

This study evaluated physical fitness, mindfulness (MAAS-A), pain and BMI in relation to stress (PSQ) in Norwegian adolescents. Additional physical fitness associations were also explored as well as gender differences. Analyses revealed that physical fitness did not explain any variation in stress among the adolescents. However, there were some negative associations between one of the stress factors (lack of joy) and physical fitness components at a group level, which might have been influenced by conditions not measured in this study. As opposed to physical fitness, mindfulness qualities (MAAS-A), and to some degree gender, seemed to explain variation in stress among the adolescents. Physical fitness was unrelated to mindfulness (MAAS-A) but seemed somewhat related to BMI, particularly in males. Among the females, higher physical fitness (in terms of endurance) seemed related to fewer pain sites. The cross-sectional design did not allow us to determine any causal direction among the variables. Further studies might be needed to confirm these findings.

## Author contributions

BØ has made substantial contributions to the conception and design of the work, the acquisition, analysis, and interpretation of data for the work. BØ has also drafted the work, revised it critically for important intellectual content, and approved the final version to be published. BØ agrees to be accountable for all aspects of the work in ensuring that questions related to the accuracy or integrity of any part of the work are appropriately investigated and resolved. HS and MH has made contributions to the conception and design of the work, and has contributed in revising it, and given final approval of the version to be published. HS agrees to be accountable for all aspects of the work in ensuring that questions related to the accuracy or integrity of any part of the work are appropriately investigated and resolved.

### Conflict of interest statement

The authors declare that the research was conducted in the absence of any commercial or financial relationships that could be construed as a potential conflict of interest.
